# Rigorous evaluation needed to advance coaching in the biomedical workforce

**DOI:** 10.3389/fmed.2026.1830734

**Published:** 2026-07-10

**Authors:** Monica Kowalczyk, Erika Temprano, Vineet M. Arora, Jessica Himstedt, Shikha Jain, Valerie G. Press, Alexandra Tate, Rachel K. Wolfson, Anna Volerman

**Affiliations:** 1 Department of Medicine, University of Chicago, Chicago, IL, United States; 2 Pritzker School of Medicine, University of Chicago, Chicago, IL, United States; 3 Insight Studio, Littleton, CO, United States; 4 Department of Medicine, University of Illinois Chicago, Chicago, IL, United States; 5 Department of Pediatrics, University of Chicago, Chicago, IL, United States

**Keywords:** biomedical sciences, coaching, evaluation, faculty, professional development, research, workforce

## Abstract

Researchers in the biomedical workforce face challenges in establishing their careers, with disproportionate effects for underrepresented groups. Coaching, a form of professional development commonly adopted outside of the biomedical sciences, may be a useful and effective way to help researchers as they start and advance in their careers. In this innovative intervention, coaches guide clients from various fields and specialties to maximize their work performance for a range of goals that are tailored to the client’s needs. However, coaching is not yet a widely accepted or regularly utilized practice in academic medicine and research. Coaching may help advance researchers in their careers and reduce longstanding inequities in the scientific workforce. To ensure effectiveness, coaching programs should be evaluated longitudinally using a conceptual framework; however, such rigorous evaluation has been limited to date. The authors highlight the potential benefits of coaching and call for greater evaluation of such programs in the biomedical sciences using the Kirkpatrick Model and Social Cognitive Career Theory to inform a framework for the evaluation of biomedical coaching. Maximizing the effectiveness of coaching has important potential to reduce inequities in the biomedical workforce and ensure that science benefits the nation’s population.

## Introduction

Professional development is critical to support scientists and researchers in the biomedical workforce. Studies show that professional development, such as mentorship and skills training programs, enhances career satisfaction, research productivity, and work performance (1–6). Further, professional development can help overcome the persistent workforce inequities in biomedical research (7, 8). Such supports particularly benefit individuals in early career stages who face limited high-quality mentorship, tenure-track faculty positions, and research funding, while confronting work-life imbalances (9–12). Among this group of researchers, women and others from underrepresented backgrounds are more vulnerable to challenges such as stereotype threat and imposter syndrome (13–15). Such experiences can negatively impact their transition to research independence and advancement in their careers (10–12). Without adequate support, the biomedical research enterprise can experience lower retention rates, increased turnover costs, limited research output, and less diverse innovation in scientific fields (16, 17).

In light of these challenges, one promising approach to enhance growth among biomedical researchers is professional coaching (18). Coaching helps a client improve performance and meet their professional and/or personal goals through cognitive and behavioral changes (19–21). Coaching programs have been applied in various settings and structures, with literature describing various coaching practices and methods (22). To ensure such coaching programs are effective, evaluation is essential (23). Yet, few studies evaluate the effectiveness of coaching and its mechanisms for success (24). Further research is critical to understand the impact of coaching and its lasting effects on biomedical researchers. To strength professional coaching as individuals and organizations are investing in such programs, we examine what is known about outcomes of coaching programs and call for increased evaluation of these programs to enhance their impact on the biomedical workforce. To do so, we reviewed literature on the application and evaluation of professional coaching in the biomedical sciences and other fields to identify existing practices and inform recommendations for evaluation of the effects of such interventions in the workforce.

## Understanding coaching: definitions, distinctions, and applications

In the landscape of professional development, coaching has the potential to foster both individual and institutional progress. According to the International Coaching Federation, coaching involves “partnering with clients in a thought-provoking and creative process that inspires them to maximize their personal and professional potential” (25). Coaching can assist clients in various career domains, such as transitioning into new roles or adapting to change (26). In particular, coaches work with clients on setting goals (e.g., increasing leadership capabilities, improving decision-making skills) and overcoming challenges (e.g., time management, stress management, communication skills) (27).

Overall, coaching promotes enhanced performance through individual professional and personal growth (18, 28). One theory that can explain how coaching programs support such development is the Social Cognitive Career Theory (SCCT) (29). SCCT suggests that interactions between a person and their environment create learning experiences that influence confidence and outcomes in their careers (30, 31). Coaching programs build self-efficacy among clients by offering learning experiences (e.g., skill development, self-awareness) and social supports, thereby helping promote career advancement and persistence. Such impacts extend to organizations and institutions by positively enhancing their leadership, culture, and performance (32).

Coaching is distinct from other forms of professional development, such as mentoring and advising. Mentorship is a long-term relationship in which a mentor in the same field as the mentee shares knowledge and skills from their own experience to guide the mentee in their career (18, 33). In advising, commonly applied in academic settings, an advisor counsels an individual by giving them direction on a matter in which the advisor is an expert (18, 33). In contrast, a professional coach does not necessarily have the same work experiences or knowledge as the client. The coach helps the client understand how to learn rather than teaching the client (33). In a coaching relationship, the client is the expert upon whom the coach relies to lead the intervention (20). Notably, coaches do not apply approaches like counseling or therapy and do not promote their own opinions in their work (34). Instead, the coach reaches a deeper understanding of the client’s specific needs by asking reflective questions as well as identifying goals and actions to reach an outcome (20).

There are several applications of coaching, including peer coaching, cognitive coaching, instructional coaching, and academic coaching. Peer coaching, typically applied in academic settings, involves a dyad relationship in which individuals with similar experiences work together to improve each other’s performance (35, 36). Cognitive coaching is a process in which a coach assists the client to self-reflect and take initiative to change cognitive behaviors in their work (37). Instructional coaching involves a coach working with educators to improve classroom practices and teaching by implementing evidence-based approaches that support classroom management, content enhancement, and other instructional issues (38). In academic coaching, students, typically in higher education settings, work with a coach to build their academic skills and achieve their academic goals (39).

In each application of coaching, coaches can use a multitude of tools to understand a client’s behavior and support the client in achieving their desired outcome(s). For example, coaches can work with clients through didactic lectures, one-on-one meetings, interactive seminars, group work, project work, and/or simulation exercises (40). Assessments (e.g., 360-degree surveys/multi-source feedback, personality assessment) paired with debriefing can be useful for coaches to gather objective data for clients and tailor the coaching appropriately to the client’s needs (41–43).

To date, coaching has been commonly applied in the business and education fields, with benefits noted for both individuals and organizations. In corporations, coaching has been utilized among executives to improve leadership and organizational effectiveness, particularly at times of organizational change (44, 45). Studies on executive coaching demonstrate that leaders have increased engagement and productivity, improved people management, better relationships with managers, as well as enhanced goal setting and prioritization (44). Organizational benefits from executive coaching include strengthened teamwork, increased retention, accelerated promotions, and improved client satisfaction (44). In education, coaching has been applied to promote teacher development, whereby a coach assists educators in implementing effective classroom practices that can positively impact student achievement (46). Along with the benefits for individuals, coaching in education can also benefit organizations by creating a positive and empowering environment that promotes educators’ self-efficacy, encourages innovative approaches, and enhances team dynamics (47). Such coaching successes may be translatable to other fields.

## Realizing potential: exploring benefits of coaching in biomedical field

In the biomedical sciences, coaching has gained attention in medical education and clinical care to support the development and wellbeing of trainees as well as attending physicians/faculty (48, 49). In medical education, coaching provided by faculty aims to help trainees reach their full potential through setting goals, identifying strategies to overcome challenges, improving academic performance, and fostering professional identity (18). Literature shows that such coaching can improve self-efficacy and resilience, reduce burnout symptoms, and increase openness to performance feedback among trainees (18, 50–52). Additionally, coaching can enhance clinical skill development for trainees (53, 54). Similarly, coaching for attending physicians can support their professional development, as well as improve self-efficacy, resilience, burnout, and quality of life (55–58). Lastly, coaching for faculty can support academic scholarship (59).

Further, coaching programs have been introduced to foster workforce diversity in the biomedical sciences. One coaching program for underrepresented graduate students in biological and behavioral sciences focused on career development and professional identity, with outcomes showing high interests in research activities and pursuit of research careers (60). A similar coaching program for underrepresented Ph.D. students in biomedical sciences provided social support to students that promoted their persistence in academia (61, 62). Coaching programs have also supported underrepresented investigators in career advancement. A coaching program focused on grant writing led to a large proportion of underrepresented researchers submitting at least one grant application and being awarded funding (63). Another coaching program supported mid-career women physicians in attaining grants and promotions (64). Given that underrepresented groups in the biomedical workforce face unique challenges, including stereotype threat and imposter syndrome (13–15, 65, 66), coaching can be essential for success and retention across various career stages, thus helping reduce inequities in the scientific workforce.

## Current practices for evaluating coaching programs

Rigorous evaluation of coaching programs is necessary to determine their utility and identify areas for improvement (67). Such evaluation can help clients, coaches, and organizations to understand the various effects of coaching programs and interventions. For instance, evaluation can provide evidence to a client of their development by recording changes in performance (68). For coaches, evaluation can provide insight on how they can improve the quality of their coaching (69, 70). Lastly, evaluation can inform organizations about the return on investment of the professional development program (67, 68).

While the evaluation of coaching programs has holistic benefits, it is not commonly done. In fact, a 2007 study found only one-third of coaching programs were evaluated (71). A more updated study is not available currently; however, recent literature on coaching aligns with this study, suggesting evaluation remains inadequate (24, 38, 72). This gap may be due to the fact that professional coaching is difficult to assess with its individualized approach, diverse outcomes, and influencing factors (e.g., client attitude, relationship between coach and client, coach skills) (68, 69, 73). There is a need for a systematic approach to evaluation that collects and analyzes data to understand outcomes of professional coaching and opportunities for improvement (68, 74).

For rigorous evaluation, one framework that can support a systematic approach is the Kirkpatrick Model, a foundational framework for evaluating outcomes of training and professional development programs, including coaching (74–76). This model consists of four hierarchical levels of evaluation: reaction, learning, behavior, and results (Table 1). The first two levels target short-term outcomes. “Reaction” assesses client’s perspectives on the coaching program, including satisfaction and perceived effectiveness. “Learning” measures the extent to which clients learned from coaching, capturing cognitive changes like knowledge (e.g., awareness of strengths and weaknesses) and skill development (e.g., adaptability). This level can also assess changes in attitudes and self-efficacy. The higher two levels examine more tangible measures and often use multiple sources of data. “Behavior” assesses changes in targeted behaviors that occur due to coaching, an important factor that influences work performance. The “Results” level of the model traditionally measures the program’s impact on the organization, for example productivity, revenue, retention, and customer satisfaction or outcome. For comprehensive evaluation of coaching, each level of the model should be assessed using both quantitative and qualitative data from various individuals (e.g., client, supervisor, peer, subordinate) collected before and after programming (67, 68, 77). Additionally, it is recommended to compare coaching to a control group, in a randomized way when possible, to determine its impact and specific outcomes (76).

**TABLE 1:** Application of Kirkpatrick Model for evaluating coaching programs.

Level	Measurement	Methodology	Examples of approaches utilized in studies
Reaction	How does client feel about the program they attended? To what extent are they satisfied?	Post-program evaluation in form of: - Survey, questionnaire, and feedback forms - Verbal and written reports	- Peer coaching intervention for board-eligible/certified surgeons: after completing intervention, participating surgeons provided written evaluation of the program, including value of and satisfaction with program (57). - Longitudinal coaching program for medical students: at the end of each academic year, students completed a survey to rate the extent that their coach guided and supported them in fostering personal and professional development, advancing physician skills, and promoting wellbeing and belonging (reported on Likert scale) (53). - Longitudinal coaching program for internal medicine residents: residents completed a post-intervention survey to rate coaching experience (excellent, good, fair/poor) and if they would advise other residency programs to implement coaching (reported on Likert scale) (50).
Learning	To what extent has client learned the information and skills? To what extent have their attitudes been changed?	Pre/post-program evaluation in form of: - Surveys and questionnaires - Interviews - Knowledge tests	- Coaching program for pediatric resident physicians: resident physicians completed pre/post-program survey to assess patient communication skills and attitudes toward patient feedback (utilized validated Communication Assessment Tool to rate physician communication with a Likert scale) (54). - Longitudinal coaching program for medical students: students completed survey to determine their learning of patient care skills (patient communication, building a medical history, physical examination) and health systems skills (microsystem improvement, interprofessional collaboration) through coaching guidance (53).
Behavior	To what extent has client’s job behavior changed as a result of the program?	Pre/post-program evaluation in form of: - Assessment (written or verbal) on client’s performance and behavior reported by supervisor, peers, or subordinates - Observations and measures of behavior changes	- Coaching program for 1st year medical students: participating students completed pre/post-surveys to assess changes in resilience (with Connor-Davidson Resilient Beliefs Scale), perceived stress (with Friedricksson-Larson single question), awareness/management of stress, time management, goal setting, as well as energy for relationships and school (all reported on a Likert scale) (51). - Longitudinal coaching program for internal medicine residents: participating resident physicians completed a post-intervention survey about if the coaching program improved ability to cope with stress, distress in personal life, information processes, work-life balance, work relationships, self-confidence, and administrative burdens (50). - Coaching program for pediatric resident physicians: patients of participating resident physicians completed pre/post-feedback forms to assess communication skills (with Communication Assessment Tool), as well as what the resident physician did well and could do better in terms of communication (54). - Coaching program for physician leaders: physician leaders completed interviews 2 months after intervention in which they described in-depth the application of skills learned in coaching and the behavioral changes seen (83).
Results	To what extent have results been affected by the program?	Post-program evaluation focused on organizational performance and other return on investment indicators, including: - Cost savings - Quality ratings - Career outcomes (burnout, promotion, retention) - Customer outcomes	- Executive coaching program for senior leaders: participating executives completed post-program interviews focused on how they applied their coaching to impact productivity, work quality, diversity, retention and promotion of leadership, teamwork, client satisfaction, as well as estimated monetary benefit from coaching (84). - Coaching program for physician leadership: organization stakeholders assessed if participating physicians’ deliverables from coaching program (organization-wide team project) aligned with organization’s goals (83). - Large-scale coaching program for doctors and dentists: participating doctors and dentists completed post-program questionnaire to assess program impacts on working with patients and colleagues and on self, including intention to stay in position (85). - Coaching program for internal medicine interns: Interns completed pre/post-program survey to measure changes in emotional exhaustion (with Maslach Burnout Inventory) (52).

To date, approaches for evaluating coaching programs remain limited. Studies prioritize short-term outcomes, oftentimes immediately after the coaching program (67, 68, 78). Further, they largely focus on the reaction level, for example client satisfaction, which results in inadequate assessment on the effectiveness of coaching (67, 68, 78). Studies also heavily rely on self-reported data from clients and rarely gather data from supervisors, peers, and subordinates (68). Lastly, few evaluations of coaching programs examine long-term outcomes or explore factors that influence outcomes (e.g., coaching processes, coach-client relationships) (68). Notably, these limitations in evaluation efforts are more apparent among the coaching programs in the biomedical workforce. The majority of studies that focus on coaching in the biomedical sciences utilize self-reported data from clients, particularly quantitative data (50–52, 55, 57). Few such studies examine long-term outcomes (49, 50, 53, 59), collect multiple perspectives (53, 57), or explore coaching processes and coach-client relationships (53) –all of which are important to optimize coaching in the biomedical sciences. As such, gaps remain in well-rounded evaluation that is needed to delineate the full impact of coaching on individuals and organizations.

## Discussion: recommendations for evaluating coaching programs in biomedical field

**FIGURE 1:**
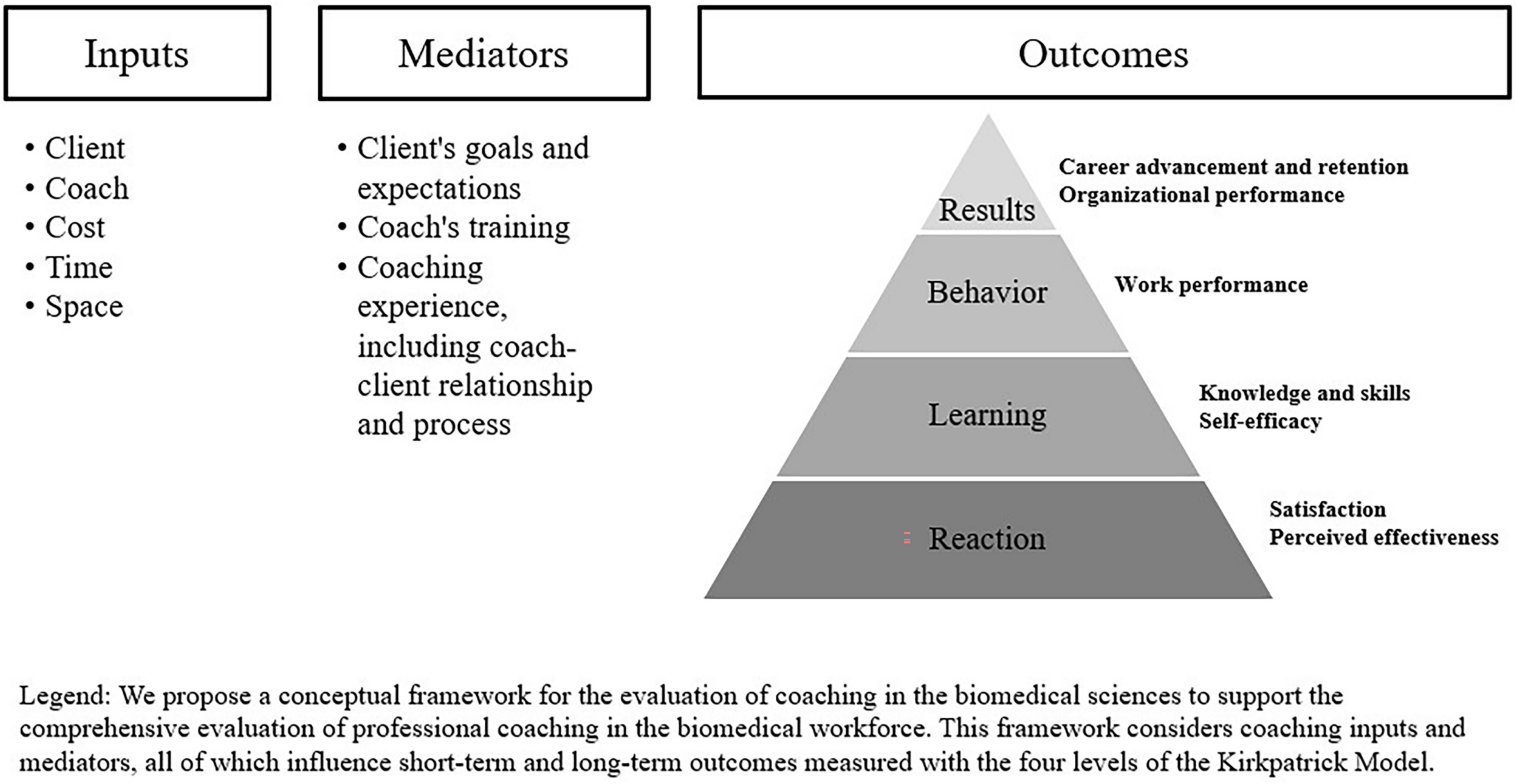
Biomedical Coaching Evaluation Framework, based on Social Cognitive Career Theory and Kirkpatrick Model. Legend: We propose a conceptual framework for the evaluation of coaching in the biomedical sciences to support the comprehensive evaluation of professional coaching in the biomedical workforce. This framework considers coaching inputs and mediators, all of which influence short-term and long-term outcomes measured with the four levels of the Kirkpatrick Model.

To ensure coaching programs in the biomedical sciences are viable and optimal, evaluation must be comprehensive, including using a conceptual framework to examine outcomes across varied levels and perspectives. We propose the Biomedical Coaching Evaluation Framework, a conceptual framework for the evaluation of coaching in the biomedical sciences based on SCCT and the Kirkpatrick Model, which considers coaching input, mediators, and outcomes (Figure 1). Evaluation efforts should incorporate all four levels of the Kirkpatrick Model to determine both short-term and long-term effects of coaching on clients and organizations.

Longitudinal studies can assess distal outcomes, for instance the broader effects of coaching at the organizational level like productivity, efficiency, retention, organizational climate, and return on investment (67, 68). Other considerations for long-term outcomes to integrate into evaluation include client-focused metrics like burnout, promotion, retention, compensation, and career satisfaction (67, 79). Given that it can be challenging to attribute long-term outcomes to a particular experience, studies should also collect data on other factors that may influence clients’ career outcomes, such as additional professional development opportunities or life events, which can be used to help isolate the effects of coaching. Additionally, evaluation efforts should leverage multisource data from various individuals, such as self-assessments completed by clients and 360-degree assessments completed by peers and supervisors to observe changes in the client’s performance and behavior.

In terms of evaluating factors that influence coaching outcomes, efforts can incorporate metrics focused on the success of coach-client relationship (e.g., expectation, engagement) and coaching process (e.g., goal setting, problem identification, resolution) from both client and coach perspectives (18, 68). Further, evaluation can explore the professional experiences and skills among coaches to identify competencies and trainings that are most effective for coaching of the biomedical workforce. Such efforts are particularly important given that several organizations provide various training and certifications for professional coaching (80).

Collectively, such data can be a key part of a mixed-methods approach to evaluation, which is proven to support a comprehensive understanding of coaching outcomes (81, 82). Additionally, studies designed as randomized controlled trials can further delineate outcomes specific to coaching.

As coaching expands in the biomedical sciences field, it will be valuable to examine the effects of coaching programs on diversity and inclusion in the workforce. Coaching may impact individuals differently, as each client has unique experiences in their profession, particularly those who are underrepresented in the field. For coaching programs to be effective, they must be relevant and adapted to diverse professional and cultural backgrounds of clients. Evaluation can support such tailoring of programs. Further, examining the effects of professional coaching based on gender identity, sexual orientation, race/ethnicity, disability, and other areas, as well as assessing for differential effects by groups, can inform both coaches and organizations about how to best support diverse clients. Such evaluation can strengthen coaching to address the unique barriers that professionals face in the biomedical workforce.

## Conclusion

Research shows that coaching can be a promising approach for advancing professional development in the biomedical workforce. The benefits of coaching in various fields like business and education are well-documented in literature. However, research on coaching in the biomedical field is underdeveloped, lacking comprehensive evaluation to determine individual and program outcomes. Current efforts focus on short-term outcomes of coaching and rely on self-reported data from clients. Future evaluation of coaching programs in the biomedical sciences should apply a conceptual framework, examine long-term outcomes and leverage multisource data, particularly among clients of diverse backgrounds. With such rigorous evaluation, coaching can be designed to optimally support professionals as well as improve diversity and inclusion within the biomedical workforce.

## Data Availability

The original contributions presented in this study are included in this article/supplementary material, further inquiries can be directed to the corresponding author.
